# Mesoscopic Moment Equations for Heat Conduction: Characteristic Features and Slow–Fast Mode Decomposition

**DOI:** 10.3390/e20020126

**Published:** 2018-02-15

**Authors:** Luca Bergamasco, Matteo Alberghini, Matteo Fasano, Annalisa Cardellini, Eliodoro Chiavazzo, Pietro Asinari

**Affiliations:** Energy Department, Politecnico di Torino, Corso Duca degli Abruzzi 24, 10129 Turin, Italy

**Keywords:** heat conduction, mesoscopic models, kinetic theory, Cattaneo equation, Extended Irreversible Thermodynamics

## Abstract

In this work, we derive different systems of mesoscopic moment equations for the heat-conduction problem and analyze the basic features that they must hold. We discuss two- and three-equation systems, showing that the resulting mesoscopic equation from two-equation systems is of the telegraphist’s type and complies with the Cattaneo equation in the Extended Irreversible Thermodynamics Framework. The solution of the proposed systems is analyzed, and it is shown that it accounts for two modes: a slow diffusive mode, and a fast advective mode. This latter additional mode makes them suitable for heat transfer phenomena on fast time-scales, such as high-frequency pulses and heat transfer in small-scale devices. We finally show that, if proper initial conditions are provided, the advective mode disappears, and the solution of the system tends asymptotically to the transient solution of the classical parabolic heat-conduction equation.

## 1. Introduction

Over the last decades, considerable efforts have been spent on the goal to extend the phenomenological concept of irreversible thermodynamics into the region beyond the classical hydrodynamic description [1]. There are several reasons for this interest. One of these is to overcome the limits of the classical transport equations to correctly describe high-frequency and short-wavelength processes [2,3,4]. This deficiency has become particularly limiting during the last years because of the increasing interest in small-scale devices, nano-technologies and nano-structured materials [5,6,7,8,9,10]. Contemporary technology strives for high speed and miniaturization; thus, transport equations should be able to cope with the related phenomena. Another reason, perhaps even more important, is the unphysical behavior of the classical parabolic partial differential equations, which imply that perturbations propagate with an infinite speed. This is not only disturbing from a theoretical point of view, but it is also incompatible with experimental observations [11,12]. As a typical case, the heat-conduction problem has been extensively discussed. The classical heat-conduction equation is based on Fourier’s law as follows [13]:

(1) q=−λ∇T

where the heat flux **q** is directly related to the temperature gradient through the thermal conductivity λ. This equation successfully recovers experimental data over a wide range of engineering applications of practical interest; however, it fails for fast transients and presents the unpleasant property to predict an instantaneous temperature propagation. It lacks indeed inertial effects associated with the acceleration of the heat flow, which is sometimes called *dissipative flux*. In order to overcome this limit, Cattaneo [14] proposed a damped version of Fourier’s law in the form

(2) $$q+\tau \frac{\partial q}{\partial t}=-\lambda \nabla T$$

where the time constant τ in the additional heat-flux relaxation term accounts for the response time of the heat flow when a sudden temperature gradient is imposed. This celebrated equation has had an enormous influence on the subsequent developments of non-equilibrium thermodynamics, paving the way for more sophisticated models. Few years later indeed, Guyer & Krumhansl [15,16] proposed an extended model for diffusive-hyperbolic heat conduction on the basis of the solution of the linearized phonon Boltzmann equation. The model is established on kinetic theory foundations and has the merit to emphasize the role of non-local effects on heat transport; notwithstanding, the resulting temperature equation is parabolic, and, thus, it predicts the propagation of thermal signals at infinite speed. An interesting critical review of this model and a comparison with the Cattaneo equation can be found in [17]. The surge of interest in a more complete description of heat conduction with finite speed of propagation led to further investigations of the Cattaneo equation [18,19,20,21] and to a number of models and approaches to the problem (see [22] for a complete review), encompassing the realm of rational thermodynamics [23,24,25].

In this work, we focus on kinetic approaches, which have been extensively used to obtain new heat-conduction equations derived from the Boltzmann equation [26,27,28,29] and to investigate regimes in which multiple scales and sub-continuum effects are important [30,31,32]. On the basis of the procedure reported in [33], we derive different systems of mesoscopic moment equations for heat conduction, discussing the basic properties that they must hold. We then show that, considering two-equation systems, the resulting mesoscopic equation for temperature complies with the Cattaneo equation in the Extended Irreversible Thermodynamics Framework. The analytical solution of the proposed systems shows that they are able to account for two scales: a slow, diffusive time-scale and a fast, advective time-scale. We also show that, for proper initial conditions, the system reduces to the purely diffusive case, recovering the classical parabolic heat-conduction equation.

Thus, the proposed models provide an extended description that is able to account for heat transfer phenomena occurring at fast time-scales, which are not recovered by Fourier’s description. Practical applications for these models, under proper considerations, span over nano-technologies and -materials in a wide sense, for which characteristic modeling sizes typically approach the mean free path or wavelength of the energy or information carriers [34]. Example engineering applications include thermoelectric nanomaterials [35] for modeling heat transport in microelectronic devices, passive cooling and thermoelectric energy conversion [36]; and nano-engineered suspensions for radiative cooling [37], volumetric solar thermal energy absorption [38], solar desalination [39] and nano-actuators [40]. Applications can be also found in the biomedical sector, such as in medical treatments, where the temperature of the nano-constructs injected in the treated tissues can be controlled to perform cryosurgery [41] or treat hyperthermia [42]. Non-Fourier phenomena also appear in localized and pulsed heat transfer through skin tissues and blood vessels, where non-linear dual-phase-lag models are typically used [43].

The paper is organized as follows. In Section 2, we introduce and solve analytically the macroscopic reference equation, that is, the classical parabolic heat-conduction equation, using Fourier transforms. We then introduce the mesoscopic systems of moments in Section 3, discussing two-equation versions and a three-equation version. In Section 4, we obtain the characteristic frequencies of the proposed systems, and we present the mode-decomposition analysis in Section 5. Finally, in Section 6, we provide a discussion on the results obtained, and we draw the final conclusions in Section 7.

## 2. Macroscopic Description

### 2.1. Parabolic Heat Conduction: Infinite Harmonics

Substituting Fourier’s law of Equation (1) into the energy balance equation written for a system at rest and in the absence of source terms, a parabolic partial differential equation for the temperature evolution is obtained [1]. Considering the one-dimensional case for simplicity, in the real positive domain, it reads as

(3) $$\frac{\partial T}{\partial t}=\alpha \frac{\partial^{2}T}{\partial x^{2}}$$

where T=T(x,t) is the temperature and $\alpha =\lambda /(\rho c_{p})$ is the thermal diffusivity (with ρ the density and $c_{p}$ the specific heat). We assume the temperature field to be absolutely integrable over the space domain considered and define the Fourier transform pair as follows [44]:

(4) $$\begin{array}{c} \hat{T}\left(k,t\right)=\int_{-\infty }^{+\infty }T\left(x,t\right)e^{-ikx}dx\;\mathrm{and}\;T\left(x,t\right)=\frac{1}{2\pi }\int_{-\infty }^{+\infty }\hat{T}\left(k,t\right)e^{ikx}dk \end{array}$$

Thus, Equation (3) can be rewritten as follows:

(5) $$\begin{array}{c} \frac{\partial }{\partial t}\int_{-\infty }^{+\infty }\hat{T}\left(k,t\right)e^{ikx}dk=-\alpha \int_{-\infty }^{+\infty }k^{2}\hat{T}\left(k,t\right)e^{ikx}dk \end{array}$$

Deriving by *k*, the integrals simplify and, considering *k* as a parameter, we obtain

(6) $$\frac{d\hat{T}}{dt}=-\alpha k^{2}\hat{T}$$

Thus, using the Fourier transform pair, we have mapped the heat equation to an ordinary differential equation, whose general solution is

(7) $$\hat{T}\left(k,t\right)=\hat{T}\left(k,0\right)e^{-\alpha k^{2}t}$$

Letting the initial condition be in the form $T\left(x,0\right)=\sin\left(k_{0}x\right)$, the solution yields

(8) $$\hat{T}\left(k,t\right)=i\pi \left[\delta \left(k+k_{0}\right)-\delta \left(k-k_{0}\right)\right]e^{-\alpha k^{2}t}$$

**Figure 1:**
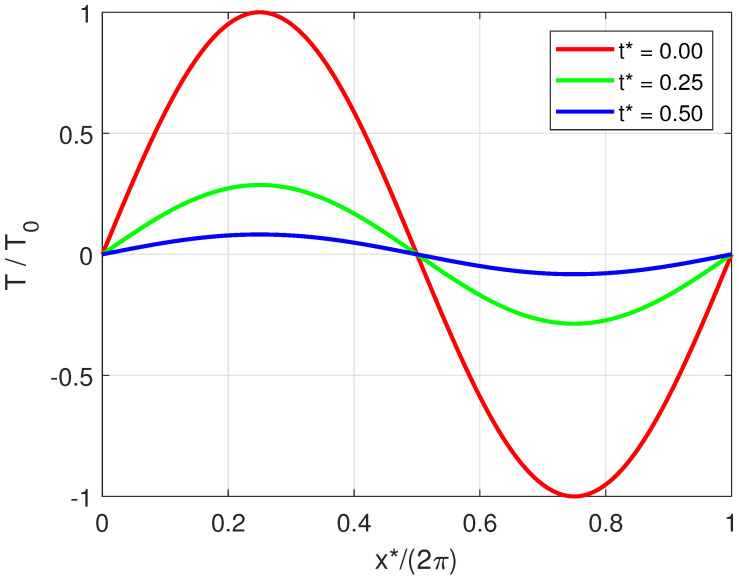
Solution of the macroscopic heat conduction given by Equation (9) as a function of the dimensionless coordinate $x^{*}=xk$ and time $t^{*}=tck$.

The solution with respect to the physical temperature *T* can be easily obtained via the inverse Fourier transform as follows:

(9) $$T\left(x,t\right)=\sin\left(k_{0}x\right)e^{-\alpha k_{0}^{2}t}$$

which is the well-known solution of the purely diffusive, parabolic heat-conduction equation. In this case, the temperature T(x,t) shows a sinusoidal trend with respect to space and an exponential decay with relaxation rate $\tau =1/(\alpha k_{0}^{2})$ with respect to time. The solution is shown in Figure 1.

In order to obtain the solution given by Equation (9), we used the Fourier transform and inverse transform. The same result can be obtained by introducing a temperature in the complex domain and using the inverse Fourier transform only. For this purpose, Equation (3) is rewritten as

(10) $$\int_{-\infty }^{+\infty }\frac{\partial }{\partial t}\hat{T}\left(k,t\right)e^{ikx}dk=\alpha \int_{-\infty }^{+\infty }\frac{\partial^{2}}{\partial x^{2}}\hat{T}\left(k,t\right)e^{ikx}dk$$

Deriving by *k* and letting the complex temperature be $\hat{T}\left(k,t\right)e^{ikx}=\Theta \left(k,x,t\right)$ yields

(11) $$\frac{\partial \Theta }{\partial t}=\alpha \frac{\partial^{2}\Theta }{\partial x^{2}}$$

which is in fact the heat-conduction equation in the complex domain, that is, Θ∈C, whereas the real temperature $T\in R^{+}$. Analogously to Equation (7), the solution of Equation (11) yields

(12) $$\Theta \left(k,x,t\right)=\hat{T}\left(k,0\right)e^{\left(ikx-\alpha k^{2}t\right)}=\Theta_{0}e^{i(kx+\omega t)}$$

where $\omega =i\alpha k^{2}$. The physical temperature can be recovered using the inverse Fourier transform:

(13) $$T\left(x,t\right)=\frac{1}{2\pi }\int_{-\infty }^{+\infty }\Theta \left(k,x,t\right)\;dk=\frac{1}{2\pi }\int_{-\infty }^{+\infty }\hat{T}\left(k,t\right)e^{ikx}dk$$

### 2.2. Parabolic Heat Conduction: Finite Harmonics

In the previous section, we have demonstrated that, in general, the temperature in the real domain can be obtained via the inverse Fourier transform of the complex temperature. From a discrete point of view, the integral can be approximated using a quadrature formula:

(14) $$T\left(x,t\right)=\frac{1}{2\pi }\sum_{k_{n}=1}^{\infty }\Theta_{0k}e^{i(k_{n}x+\omega t)}$$

where *k* is the wavenumber and depends on the number *n* of the harmonic to which we are referring. For any given value of $k_{n}$, we obtain the same form of the solution of Equation (9). This can be easily demonstrated by the substitution of Equation (12) into Equation (11), which yields $\omega =i\alpha k^{2}$. In the following, we analyze a generic single harmonic for $k_{0}$, but the analysis can be easily extended for multiple harmonics. In this latter case, any harmonic would have the same form but a different fundamental pulsation, and the general solution would be the sum of the harmonics considered.

## 3. Mesoscopic Description

### 3.1. Modeling Approach and Physical Background

In this work, our purpose is to (i) provide alternative approaches to the bottom-up expansion from kinetic theory (see Section 1) for the development of mesoscopic systems, and (ii) provide a systematic methodology for the analytical analysis of these systems. The latter is particularly meant to be useful, under proper considerations, for understanding more complex cases and, to our knowledge, has not been reported before in the proposed detail. Thus, in the following sections, we develop mesoscopic systems for the heat-conduction problem starting from Fourier’s description and using a top-down approach, analyzing their solution in detail.

A mesoscopic description of a transport process requires a number of partial differential equations greater than that of the macroscopic description; in this work, we discuss two- and three-equation systems. We first introduce the physical background of the modeling approach. This background is discussed on the basis of the flow regimes, which are classified according to the value of the Knudsen number. This number, which we indicate with ϵ in this work, is generally defined as the ratio of the mean-free path of the fluid particle to the characteristic length scale of the fluid flow. The following classification for the flow regimes holds [33]:

- Hydrodynamic regime for ϵ≤0.01: This can be described using macroscopic continuum models, for example, the Navier–Stokes–Fourier (NSF) system of equations. With regard to heat transfer, the standard applications in this regime involve the thermal analysis of macroscopic engineering devices.
- Slip-flow regime for 0.01<ϵ≤0.1: this can still be described using the NSF system, but additional boundary conditions must be taken into account to correctly describe the slip velocity and temperature jumps at the interfaces. In particular, example applications of temperature jumps (or Kapitza discontinuities) involve the analysis of the heat transfer in nano-particle suspensions at the interface between a solvent and solute.
- Transition regime for 0.1<ϵ≤10: the NSF equations are no longer valid, and a more complete approach must be used to correctly describe the fluid flow. In this regime, extended equations for higher-order hydrodynamics must be adopted, and the thermal transport mechanisms transition from diffusive, ϵ≪1, to ballistic, ϵ≫1. Example applications of thermal transport in this regime include hyperbolic heat transfer, for which there is a finite speed of energy transfer.
- Free molecular flow for ϵ>10: this is dominated by particle-wall collisions and must be described using a molecular level of detail. Typical applications in this regime include the analysis of phonon transport in nano-structures and -aggregates.

Kinetic theory provides the means to bridge discrete and continuum approaches, that is, to address the slip-flow and the transition regime. In this work, we focus on Knudsen numbers in the range 0.01<ϵ<0.1; therefore, we span from the hydrodynamic regime to the full slip-flow regime (or very early transition regime). For the sake of simplicity, we consider a one-dimensional domain with periodic boundary conditions.

### 3.2. First Mesoscopic System: Two-Moment Hyperbolic Equations

In order to show how to build a first example of a mesoscopic equation, we multiply Equation (3) by the parameter ϵ squared; this yields

(15) $$\epsilon^{2}\frac{\partial T}{\partial t}+\epsilon \frac{\partial }{\partial x}\left(-\epsilon \alpha \frac{\partial T}{\partial x}\right)=0$$

Recalling that the heat flux is given by Fourier’s law of Equation (1) as q=−λ∂T/∂x and that the thermal diffusivity is $\alpha =\lambda /(\rho c_{p})$, the above equation can be rewritten in the following form:

{(16a)ϵ2∂T∂t+ϵρcp∂φ∂x=0(16b)ϵα∂T∂x=−1ρcpφ

where the new variable φ=ϵq is called the *ghost moment* and does not appear in the macroscopic model. The concept of moments comes from kinetic theory, where particle dynamics is statistically described using distribution functions. In this framework, macroscopic quantities are obtained from the moments of the particle distribution functions; thus, macroscopic equations can be recovered from the statistical description—that is, the Boltzmann transport equation—using asymptotic expansion techniques, such as Hilbert or Chapman–Enskog expansions [33]. The ghost moments are those that have a higher order with respect to those required to recover the target hydrodynamic level (hydrodynamic moments). Taking into account these moments provides a richer description of the physical phenomenon; however, they also lead to a more complex mathematical framework. In this case, we start from the Fourier description and add a ghost moment, which has units of thermal flux, to increase the order of the physical description. However, Equations (16a) and (16b) do not represent a mesoscopic model yet, even for ϵ→∞, as for each value of the variable ϵ, the system recovers Equation (3) by substitution. Hence, we refer to the following system of equations:

{(17a)ϵ2∂T∂t+ϵρcp∂φ∂x=0(17b)ϵ2αρcpc2∂φ∂t+ϵα∂T∂x=−1ρcpφ

where *c* is a velocity. This velocity is a constant, and, in this work, its value is defined arbitrarily for methodological purposes; however, in practical applications, it depends on the properties of the considered material. Because we are building the system from purely diffusive heat conduction, meaning that no advection takes place, the velocity must be an input parameter of the model. The additional term involving the time derivative of the ghost moment is responsible for the enriched mesoscopic description. Equations (17a) and (17b) indeed represent a first example of a mesoscopic system, which in the following we name the Two-Moment Hyperbolic Equation (MESO1) system. We also notice that the MESO1 system is now hyperbolic, which is consistent with an extended thermodynamic description of finite-time heat-diffusion processes. Mesoscopic methods tend indeed to kinetic theory and thus to a strictly hyperbolic framework. We note that the smaller the Knudsen number ϵ (eventually ϵ→0), the more the problem lends itself to a macrocopic description. This means that, for small values of ϵ, the mesoscopic model approaches the macroscopic model. We now write the mesoscopic system as a single equation. From Equation (17a), we obtain

(18) $$\frac{1}{\rho c_{p}}\frac{\partial \varphi }{\partial x}=-\epsilon \frac{\partial T}{\partial t}$$

Deriving Equation (17b) with respect to space and replacing Equation (18) yields

(19) $$\frac{1}{\rho c_{p}}\frac{\partial \varphi }{\partial x}=\frac{\alpha \epsilon^{3}}{c^{2}}\frac{\partial^{2}T}{\partial t^{2}}-\epsilon \alpha \frac{\partial^{2}T}{\partial x^{2}}$$

Using again Equation (18), we obtain the following mesoscopic equation in terms of real temperature:

(20) $$\frac{\alpha \epsilon^{2}}{c^{2}}\frac{\partial^{2}T}{\partial t^{2}}+\frac{\partial T}{\partial t}=\alpha \frac{\partial^{2}T}{\partial x^{2}}$$

which can be also written in terms of the complex temperature as

(21) $$\frac{\alpha \epsilon^{2}}{c^{2}}\frac{\partial^{2}\Theta }{\partial t^{2}}+\frac{\partial \Theta }{\partial t}=\alpha \frac{\partial^{2}\Theta }{\partial x^{2}}$$

### 3.3. Second Mesoscopic System: Switched Two-Moment Hyperbolic Equations

The procedure we have used to obtain the mesoscopic equation in the previous section for MESO1 is not unique. We can in fact define a second type of mesoscopic system as

{(22a)ϵ2c2∂φ∂t+ϵρcp∂T∂x=0(22b)ϵ2∂T∂t+ϵρcp∂φ∂x=−c2αT

where the velocity now appears in both equations. In order to write the system as a single mesoscopic equation, we proceed similarly to the previous section. From Equation (22a), we have

(23) $$\frac{\partial \varphi }{\partial t}=-\rho c_{p}\frac{c^{2}}{\epsilon }\frac{\partial T}{\partial x}$$

Deriving Equation (22b) with respect to time and substituting Equation (23), we obtain the mesoscopic equation that we name the Switched Two-Moment Hyperbolic (MESO2) system:

(24) $$\frac{\alpha \epsilon^{2}}{c^{2}}\frac{\partial^{2}T}{\partial t^{2}}+\frac{\partial T}{\partial t}=\alpha \frac{\partial^{2}T}{\partial x^{2}}$$

The above equation is identical to Equation (20); thus, we are able to claim that both the MESO1 and MESO2 systems provide the same mesoscopic equation (both in the real and complex domains).

### 3.4. Third Mesoscopic System: Three-Moment Hyperbolic Equations

Until now, we have defined mesoscopic systems using two equations; it is also possible to develop mesoscopic models considering three equations. For this purpose, we consider the system of Equations (16a) and (16b); we introduce a new ghost moment *e* and, by analogy with Equations (17a) and (17b), write

{(25a)ϵ2∂T∂t+ϵρcp∂φ∂x=0(25b)ϵ2αρcpc2∂φ∂t+ϵα∂e∂x=−1ρcpφ

If *e* has the same units of temperature and is defined such that e−T=0, then Equation (25b) is equal to Equation (17b), and the system reduces to MESO1. We introduce a third equation, so that the mesoscopic system, namely, the Three-Moment Hyperbolic Equation (MESO3) system, takes the following form:

{(26a)ϵ2∂T∂t+ϵρcp∂φ∂x=0(26b)ϵ2αρcpc2∂φ∂t+αϵ∂e∂x=−1ρcpφ(26c)ϵ2∂e∂t+ϵρcp∂φ∂x=1γTθ−e

where γ and θ are new parameters, having units of time and dimensionless, respectively. The new term on the right-hand side of the third equation of the system is a forcing term, which allows us to tune the thermalization of the system. We note that if θ=1, the MESO3 system reduces to MESO1. As for the previous cases, the system can be rewritten in the complex domain, defining the complex value for the ghost flux as $\Phi ={\hat{\varphi }}^{\;ikx}$ and that for the new moment *e* as $\Psi ={\hat{e}}^{\;ikx}$. We obtain

{(27a)ϵ2∂Θ∂t+ϵρcp∂Φ∂x=0(27b)ϵ2αρcpc2∂Φ∂t+αϵ∂Ψ∂x=−1ρcpΦ(27c)ϵ2∂Ψ∂t+ϵρcp∂Φ∂x=1γΘθ−Ψ

It is possible now to rewrite the mesoscopic system using a single equation. To this end, we consider the system in the real domain; Equation (26c) is derived with respect to space and Equation (26b) is used to obtain

(28) $$-\epsilon^{2}\frac{\partial }{\partial t}\left(\frac{1}{\rho c_{p}}\varphi +\frac{\alpha \epsilon^{2}}{\rho c_{p}c^{2}}\frac{\partial \varphi }{\partial t}\right)+\frac{\alpha \epsilon^{2}}{\rho c_{p}}\frac{\partial^{2}\varphi }{\partial x^{2}}=\frac{1}{\gamma }\left(\frac{\alpha \epsilon }{\theta }\frac{\partial T}{\partial x}+\frac{1}{\rho c_{p}}\varphi +\frac{\alpha \epsilon^{2}}{\rho c_{p}c^{2}}\frac{\partial \varphi }{\partial t}\right)$$

The previous equation is derived with respect to space, and Equation (26a) is used to obtain

(29) $$\frac{\partial^{2}T}{\partial t^{2}}\left(\gamma \epsilon^{2}+\frac{\alpha \epsilon^{2}}{c^{2}}\right)+\frac{\partial T}{\partial t}=\frac{\alpha }{\theta }\frac{\partial^{2}T}{\partial x^{2}}+\gamma \alpha \epsilon^{2}\left(-\frac{\partial^{2}}{\partial x^{2}}\left(\frac{\partial T}{\partial t}\right)+\frac{\epsilon^{2}}{c^{2}}\frac{\partial^{3}T}{\partial t^{3}}\right)$$

In this case, the mesoscopic description obtained is of higher order than that of the previous cases. We note that if γ=0 and θ=1, the mesoscopic description of Equation (29) simplifies and recovers Equations (20) and (24), that is, the description obtained in the previous cases.

### 3.5. Recovering the Cattaneo Equation

It is interesting to note that the mesoscopic equations obtained from the two-equation systems, that is, Equations (20) and (24), are of the telegraphist’s type and have the same exact form of the Cattaneo equation written in terms of only the temperature [1], namely,

(30) $$\tau \frac{\partial^{2}T}{\partial t^{2}}+\frac{\partial T}{\partial t}=\alpha \frac{\partial^{2}T}{\partial x^{2}}$$

where the heat-flux relaxation $\tau =\alpha \epsilon^{2}/c^{2}$. We have also seen that an extended mesoscopic description beyond Cattaneo’s description has been achieved from the three-equation system. However, if higher-order terms are eliminated, for example, assuming γ=0 and θ=1, the Cattaneo equation is recovered. In these cases, the Cattaneo Equation (2) in terms of heat flux can be easily retrieved from Equation (30). To this end, we multiply this equation by the density ρ times the specific heat $c_{p}$, and recall that the thermal diffusivity is defined as $\alpha =\lambda /(\rho c_{p})$. This yields

(31) $$\tau \frac{\partial }{\partial t}\left(\rho c_{p}\frac{\partial T}{\partial t}\right)+\rho c_{p}\frac{\partial T}{\partial t}=\lambda \frac{\partial^{2}T}{\partial x^{2}}$$

Recalling the heat equation for the one-dimensional case:

(32) $$\rho c_{p}\frac{\partial T}{\partial t}=-\frac{\partial q}{\partial x}$$

and substituting into Equation (31), we obtain

(33) $$-\tau \frac{\partial }{\partial t}\left(\frac{\partial q}{\partial x}\right)+\rho c_{p}\frac{\partial T}{\partial t}=\lambda \frac{\partial^{2}T}{\partial x^{2}}$$

Finally, integrating in dx yields

(34) $$q+\tau \frac{\partial q}{\partial t}=-\lambda \frac{\partial T}{\partial x}$$

that is, the Cattaneo Equation (2) in the one-dimensional case.

## 4. Solution Analysis

### 4.1. Two-Equation Systems: MESO1 and MESO2

In Section 3, we have demonstrated that the mesoscopic equation of MESO1 is identical to that of MESO2; thus, we consider, for example, the MESO1 system. We recall that Θ is the complex temperature and $\Phi ={\hat{\varphi }}^{\;ikx}$ is the ghost flux. The system written in matrix notation reads

(35) $$\left[\begin{array}{cc} \epsilon^{2} & 0\\ 0 & \frac{\alpha \epsilon^{2}}{\rho c_{p}c^{2}} \end{array}\right]\left\{\begin{array}{c} \frac{\partial \Theta }{\partial t}\\ \frac{\partial \Phi }{\partial t} \end{array}\right\}+\left[\begin{array}{cc} 0 & \frac{\epsilon }{\rho c_{p}}\\ \epsilon \alpha & 0 \end{array}\right]\left\{\begin{array}{c} \frac{\partial \Theta }{\partial x}\\ \frac{\partial \Phi }{\partial x} \end{array}\right\}+\left[\begin{array}{cc} 0 & 0\\ 0 & \frac{1}{\rho c_{p}} \end{array}\right]\left\{\begin{array}{c} \Theta \\ \Phi \end{array}\right\}=\left\{\begin{array}{c} 0\\ 0 \end{array}\right\}$$

Assuming for the ghost moment Φ the same form of the solution as for the complex temperature Θ of Equation (12), the general solutions for the system are

{(36a)Θ(k,x,t)=Θ0eikx+ωt(36b)Φ(k,x,t)=Φ0eikx+ωt

Deriving the above solutions and substituting into Equation (35) yields an eigenvalue problem whose characteristic polynomial is

(37) $$\frac{\alpha \epsilon^{2}}{c^{2}}\omega^{2}-i\omega -\alpha k^{2}=0$$

The roots of the previous characteristic equation are

(38) $$\omega_{1,2}=\frac{ic^{2}\pm \sqrt{-c^{4}+4\alpha^{2}\epsilon^{2}k^{2}c^{2}}}{2\alpha \epsilon^{2}}$$

The solutions can be written in compact form as

(39) $$\left\{\begin{array}{c} \Theta \\ \Phi \end{array}\right\}=\left\{\begin{array}{c} \Theta_{01}\\ \Phi_{01} \end{array}\right\}e^{i(kx+\omega_{1}t)}+\left\{\begin{array}{c} \Theta_{02}\\ \Phi_{02} \end{array}\right\}e^{i(kx+\omega_{2}t)}=\sum_{n=1}^{2}m_{n}e^{i(kx+\omega_{n}t)}$$

and the *n*th solution $\omega_{n}$ can be substituted to recover the *n*th eigenvector $m_{n}$:

(40) $$m_{n}\left(\omega_{n}\right)=\Theta_{0n}\left\{\begin{array}{c} 1\\ -\epsilon \rho c_{p}\omega_{n}/k \end{array}\right\}$$

### 4.2. Three-Equation System: MESO3

In order to solve the MESO3 system, similarly to what we have done in the previous section, we consider the general solutions for Equations (27a), (27b) and (27c) to be in the form

{(41a)Θ(k,x,t)=Θ0ei(kx+ωt)(41b)Φ(k,x,t)=Φ0ei(kx+ωt)(41c)Ψ(k,x,t)=Ψ0ei(kx+ωt)

Deriving the above solutions and substituting into the system yields an eigenvalue problem whose characteristic polynomial is

(42) $$\epsilon^{2}i\omega \left(\frac{i\omega \alpha \epsilon^{2}+c^{2}}{\rho c_{p}c^{2}}\;\frac{i\omega \gamma \epsilon^{2}+1}{\gamma }+\frac{\alpha \epsilon^{2}k^{2}}{\rho c_{p}}\right)-\frac{i\epsilon k}{\rho c_{p}}\left(\frac{i\alpha \epsilon k}{\gamma \theta }\right)=0$$

which can be rewritten as

(43) $$\left(\gamma \epsilon^{2}\omega -i\right)\left(\frac{\alpha \epsilon^{2}}{c^{2}}\omega^{2}-i\omega -\frac{\alpha k^{2}}{\theta }\right)=\alpha \gamma \epsilon^{2}k^{2}\omega \left(1-\frac{1}{\theta }\right)$$

For θ=1, the above equation simplifies to

(44) $$\left(\gamma \epsilon^{2}\omega -i\right)\left(\frac{\alpha \epsilon^{2}}{c^{2}}\omega^{2}-i\omega -\alpha k^{2}\right)=0$$

where the characteristic polynomial of the MESO2 system in Equation (37) appears as the second term on the left-hand side. Thus, the first two roots are given by Equation (38), while the third can be obtained from the first term on the left-hand side as

(45) $$\omega_{3}=\frac{i}{\gamma \epsilon^{2}}$$

For θ≠1, that is, in the general case for Equation (43), the roots are to be computed using cubic formulas. Here we do not develop the algebra, as it goes beyond the purpose of this work. In the next sections, we concentrate on the Cattaneo-level description.

## 5. Slow- and Fast-Mode Decomposition

We now investigate in more detail the solution of the mesoscopic systems. For the sake of simplicity, we analyze only the Cattaneo-level description; thus, we focus on the two-equation systems, that is, MESO1 and MESO2. For this purpose, we consider the general solution of Equation (36a), which, according to Equation (39), we write as

(46) $$\begin{array}{cc} \Theta (k,x,t) & =\Theta_{1}+\Theta_{2}=\Theta_{01}e^{ikx}e^{i\omega_{1}t}+\Theta_{02}e^{ikx}e^{i\omega_{2}t} \end{array}$$

We consider the roots $\omega_{1}$ and $\omega_{2}$ of Equation (38), which we rewrite as

(47) $$\omega_{1,2}=i\;\frac{c^{2}\pm c\sqrt{c^{2}-4\alpha^{2}\epsilon^{2}k^{2}}}{2\alpha \epsilon^{2}}$$

On the basis of the sign of the radicand, we distinguish between and discuss two cases.

### 5.1. Case 1: αϵk/c < 1/2

The argument of the square root is positive. We Taylor expand the roots with respect to ϵ:

{(48a)ω1=ic2αϵ2−iαk2−iα3k4c2ϵ2−i2α5k6c4ϵ4+O(ϵ6)(48b)ω2=iαk2+iα3k4c2ϵ2+i2α5k6c4ϵ4+O(ϵ6)

Using the short-hand notation $C_{i}$ and $D_{i}$ for the coefficients of the expansion terms in $\omega_{1}$ and $\omega_{2}$, respectively, we can write

{(49a)eiω1t=e−C1t/ϵ2eC2teC3ϵ2teC4ϵ4t+O(ϵ6)(49b)eiω2t=e−D1te−D2ϵ2te−D3ϵ4t+O(ϵ6)

This shows that the terms $\Theta_{1}$ and $\Theta_{2}$ are both functions of time with multiple scales:

(50) $$\Theta_{1}=\Theta_{1}\left(t/\epsilon^{2},t,\epsilon^{2}t,...\right)$$

(51) $$\Theta_{2}=\Theta_{2}\left(t,\epsilon^{2}t,...\right)$$

Focusing on the first time-scale, we notice that the leading order yields the following for the two modes: (i) a fast or *advective scale* that goes to zero very quickly, because ϵ is small; (ii) a slow or *diffusive scale* that does not depend on ϵ and recovers the diffusive behavior of the macroscopic equation. Thus, for ϵ→0, the advective scale of the mesoscopic system disappears and the model recovers the macroscopic scale. In order to show the effect of the two modes on the overall time evolution of the real temperature, we inverse Fourier transform the two complex modes. Considering the leading order only, we obtain

(52) $$\begin{array}{cc} T_{1}\left(x,t\right) & =\frac{1}{2\pi }\int_{-\infty }^{+\infty }\Theta_{01}e^{ikx}e^{i\omega_{1}t}dk=T_{1}\left(x,0\right)e^{-c^{2}/\left(\alpha \epsilon^{2}\right)t} \end{array}$$

(53) $$\begin{array}{cc} T_{2}\left(x,t\right) & =\frac{1}{2\pi }\int_{-\infty }^{+\infty }\Theta_{02}e^{ikx}e^{i\omega_{2}t}dk=T_{2}\left(x,0\right)e^{-\alpha k_{0}^{2}t} \end{array}$$

**Figure 2:**
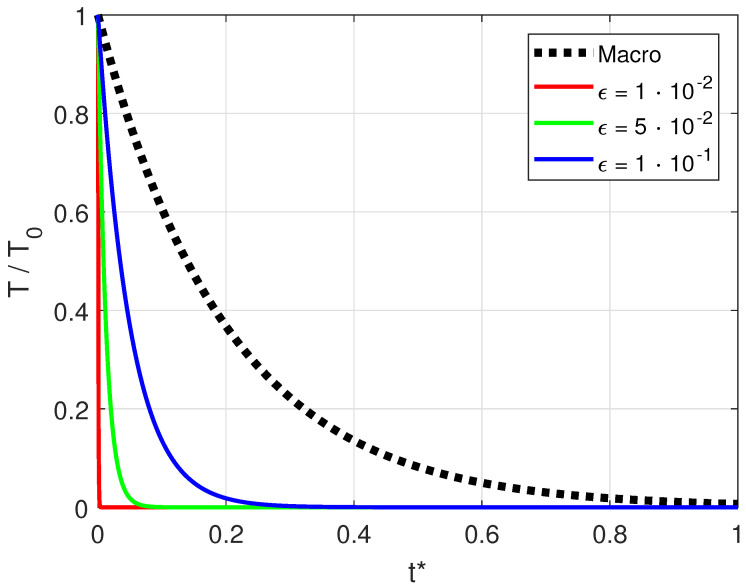
Comparison of the fast, advective mode given by Equation (49a) for different Knudsen numbers with the slow, diffusive mode, that is, Macro, given by Equation (49b). Only the leading order of the expansion of the characteristic frequencies in Equations (48a) and (48b) is shown. The dimensionless time is $t^{*}=tck$.

In Figure 2, the two modes are compared. We note that the slow, diffusive mode recovers the solution of the macroscopic model of Equation (9). The fast mode becomes relevant only for relatively high Knudsen numbers, for example, $\epsilon =10^{-1}$, while it goes to zero very quickly for small Knudsen numbers, for example, $\epsilon =10^{-2}$, thus disappearing for ϵ→0.

### 5.2. Case 2: αϵk/c > 1/2

The argument of the square root is negative; thus, the square root yields a complex number, and we expect oscillations in the solution. In order to show this behavior, we denote p=αϵk/c for compactness and rewrite the roots of Equation (38) as

(54) $$\omega_{1,2}=i\;\alpha k^{2}\frac{1\pm i\;\sqrt{1-4p^{2}}}{2p^{2}}$$

The limit of the above equations for $p\to 1/2^{+}$ yields

(55) $$\lim_{p\to 1/2^{+}}\omega_{1,2}=i\;2\alpha k^{2}$$

Thus, the time-dependent exponentials of the solutions yield

(56) $$e^{i\omega_{1}t}=e^{i\omega_{2}t}=e^{-2\alpha k^{2}t}$$

The above equation shows that αϵk/c=1/2 represents the threshold beyond which the oscillatory behavior appears and that in this condition, the two modes have the same time-scale.

### 5.3. Recovering the Single-Mode Solution

As we have previously seen, the general form of the solution for the complex temperature is given by Equation (35). Similarly, for two-equation systems, we can assume the same form for the complex heat flux. Hence, the solutions yield

(57) $$\Theta (k,x,t)=\Theta_{1}+\Theta_{2}=\Theta_{01}e^{ikx}e^{i\omega_{1}t}+\Theta_{02}e^{ikx}e^{i\omega_{2}t}$$

(58) $$\Phi (k,x,t)=\Phi_{1}+\Phi_{2}=\Phi_{01}e^{ikx}e^{i\omega_{1}t}+\Phi_{02}e^{ikx}e^{i\omega_{2}t}$$

where we notice that the solutions embed the two modes. The mesoscopic model must tend to the macroscopic model, and therefore we need to *kill* the first mode related to the root $\omega_{1}$ and keep only the second mode, which tends to $i\alpha k^{2}$ as ϵ→0. This is achieved by defining a proper initial condition that makes $\Theta_{01}$ null and thus $\Theta =\Theta_{02}$. Considering the MESO1 system, we derive and substitute the solutions of Equations (57) and (58) into the first equation of the system in Equation (35); we obtain

(59) $$\left(\epsilon \omega_{1}\Theta_{01}+\frac{k\Phi_{01}}{\rho c_{p}}\right)e^{i\omega_{1}t}+\left(\epsilon \omega_{2}\Theta_{02}+\frac{k\Phi_{02}}{\rho c_{p}}\right)e^{i\omega_{2}t}=0$$

From this, coupling the corresponding modes, we obtain

{(60a)Φ01=−ϵω1ρcpkΘ01(60b)Φ02=−ϵω2ρcpkΘ02

It can be easily demonstrated that $\Phi_{0}=\Phi_{02}$ is the proper initial condition that makes $\Theta_{01}$ null and $\Theta =\Theta_{02}$. As discussed above, this condition allows us to kill the first mode, and thus the solution of the mesoscopic system in the complex domain yields $\Theta =\Theta_{02}\exp\left(i\omega_{2}t\right)$, which can be passed to the real domain using the inverse Fourier transform of Equation (13). Hence we obtain

(61) $$T\left(x,t\right)=T\left(x,0\right)e^{i\;\omega_{2}t}=\sin\left(k_{0}x\right)\exp\left(-\frac{c^{2}-\sqrt{c^{4}-4\alpha^{2}\epsilon^{2}k_{0}^{2}c^{2}}}{2\alpha \epsilon^{2}}\;t\right)$$

**Figure 3:**
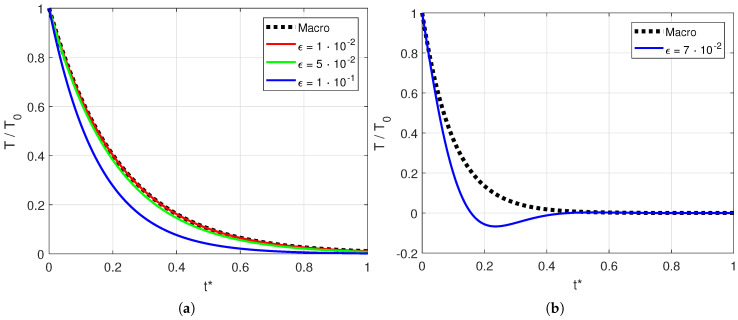
Comparison of the (single-mode) mesoscopic solution given by Equation (61) and the macroscopic solution given by Equation (9). The two analyzed cases are (**a**) $\alpha \epsilon k_{0}/c<1/2$ and (**b**) $\alpha \epsilon k_{0}/c>1/2$. The dimensionless time is $t^{*}=tck$.

In Figure 3, we compare the solution of the single-mode mesoscopic model with that of the macroscopic model. In Figure 3a, the solution for $\alpha \epsilon k_{0}/c<1/2$ is shown. We notice that for a small Knudsen number, for example, $\epsilon =10^{-2}$, the solution of the mesoscopic model recovers the macroscopic model, as expected. For larger Knudsen numbers, for example, $\epsilon =10^{-1}$, the mesoscopic model gives a different solution at short times but consistently recovers the macroscopic solution for t→∞. In Figure 3b, we show the solution for $\alpha \epsilon k_{0}/c>1/2$. We notice an oscillating trend, even for the single mode. In this case, indeed, the second mode becomes a degenerate mode, as a result of its imaginary part. This shows that, assuming constant values for α, $k_{0}$ and *c*, the variable ϵ plays an important role. We investigate in more detail the nature of these oscillations, considering, for example, $\epsilon =c/(\sqrt{2}\alpha k_{0})$. The second root yields

(62) $$\omega_{2}=\alpha k_{0}^{2}+i\;\alpha k_{0}^{2}$$

where we clearly notice the root of the macroscopic characteristic polynomial $\omega =i\;\alpha k_{0}^{2}$. Substituting Equation (62) into Equation (61), we obtain a complex quantity of which we consider the real part:

(63) $$T\left(x,t\right)=ℜ\left[\sin\left(k_{0}x\right)\exp\left(i\;\left(\alpha k_{0}^{2}+i\;\alpha k_{0}^{2}\right)t\right)\right]=\sin\left(k_{0}x\right)\cos\left(\alpha k_{0}^{2}t\right)e^{-\alpha k_{0}^{2}t}$$

Thus, the oscillations must be ascribed to the cosine function and are dumped by the exponential term; they appear indeed at short times and disappear for t→∞ as a result of the exponential decay. This result proves that the oscillations are due to the imaginary part of the solution and that they appear even in the single-mode solution if $\alpha \epsilon k_{0}/c>1/2$.

## 6. Discussion

We have demonstrated that the mesoscopic system admits a solution with multiple time-scales, for example, $\Theta =\Theta (t/\epsilon^{2},t)$, whereas the macroscopic equation admits a solution in a single time-scale Θ=Θ(t). In order to obtain further insight on this, we consider Equations (11) and (21). If the term $\alpha \partial^{2}\Theta /\partial x^{2}$, acting on ∂Θ/∂t, leads to a solution in the form Θ=Θ(t), we can assume that $\alpha \epsilon^{2}/c^{2}\;\partial^{2}\Theta /\partial t^{2}$, acting on ∂Θ/∂t, leads to $\Theta =\Theta (t/\epsilon^{2})$. Thus, Equation (21) results from the two equations:

(64) $$\begin{array}{c} \frac{\partial \Theta }{\partial t}=\alpha \frac{\partial^{2}\Theta }{\partial x^{2}} \end{array}$$

(65) $$\begin{array}{c} \frac{\alpha \epsilon }{c^{2}}\frac{\partial^{2}\Theta }{\partial t^{2}}+\frac{\partial \Theta }{\partial t}=0 \end{array}$$

We consider Equation (21) in the case of $\alpha \epsilon k_{0}/c<1/2$ and recall Equations (50) and (51). The solution can be written as the sum of two terms as follows:

(66) $$\Theta =\Theta_{1}e^{t/\epsilon^{2}}+\Theta_{2}e^{t}=\Theta \left(t_{1}=\frac{t}{\epsilon^{2}},t_{2}=t\right)$$

Computing the derivatives with respect to time and substituting into Equation (21), we obtain

(67) $$\frac{\alpha }{c^{2}\epsilon^{2}}\frac{\partial^{2}\Theta_{1}}{\partial t_{1}^{2}}+\frac{\alpha \epsilon^{2}}{c^{2}}\frac{\partial^{2}\Theta_{2}}{\partial t_{2}^{2}}+\frac{1}{\epsilon^{2}}\frac{\partial \Theta_{1}}{\partial t_{1}}+\frac{\partial \Theta_{2}}{\partial t_{2}}=\frac{\partial^{2}\Theta }{\partial x^{2}}$$

The second term on the left-hand side can be neglected as it is higher order, $O(\epsilon^{2})$. Matching terms of the same order, we obtain the following two equations for O(1) and $O(1/\epsilon^{2})$ orders:

(68) $$\begin{array}{c} \frac{\partial \Theta_{2}}{\partial t_{2}}=\alpha \frac{\partial^{2}\Theta }{\partial x^{2}} \end{array}$$

(69) $$\begin{array}{c} \frac{\alpha \epsilon }{c^{2}}\frac{\partial^{2}\Theta_{1}}{\partial t_{1}^{2}}+\frac{\partial \Theta_{1}}{\partial t_{1}}=0 \end{array}$$

The optimal initial condition sets $\Theta_{2}\gg \Theta_{1}$ and therefore $\Theta ≃\Theta_{2}$; thus, recalling that $t_{2}=t$, Equation (68) yields the macroscopic equation in the complex domain (21), from which a solution in the form Θ=Θ(t) is found. From Equation (69), recalling that $t_{1}=t/\epsilon_{2}$, we obtain

(70) $$\begin{array}{c} \frac{\alpha \epsilon^{2}}{c^{2}}\frac{\partial \Theta_{1}}{\partial t_{1}}+\Theta_{1}=0\;\Rightarrow \;\Theta_{1}=\Theta_{01}e^{-c^{2}t/\left(\alpha^{2}\epsilon^{2}\right)}=\Theta \left(\frac{t}{\epsilon^{2}}\right) \end{array}$$

This last equation confirms that the solution of the advective equation goes with the time-scale $t/\epsilon^{2}$. This result explains the reason why the mesoscopic equation has a multiple-time-scale dependence: it is essentially due to the additional term $\alpha \epsilon^{2}/c^{2}\;\partial^{2}\Theta /\partial t^{2}$.

## 7. Conclusions

In this work, we have derived different systems of mesoscopic moment equations for heat conduction. These systems have been derived starting from Fourier’s equation and using a top-down approach, as an alternative to the expansion from the kinetic framework. We have discussed mesoscopic systems based on two- and three-moment equations, showing that the former recover the Cattaneo equation, while the latter provide an increased-order description that can be reduced to the Cattaneo level under proper assumptions. The proposed systems, coherently with the kinetic framework, provide a hyperbolic description of finite-speed heat conduction. Their analytical solution has been analyzed in detail, showing that they account for two modes: a fast, advective mode and a slow, diffusive mode. The fast mode accounts for the propagation of thermal waves, which becomes relevant for time-scales on the same order of the relaxation time of the system. This is typically the case of high-frequency excitations and the small-scale modeling of thermal systems, which find applications in a number of engineering and biomedical problems. We have also shown that, if proper initial conditions are provided, the advective mode disappears, and the solution of the system tends asymptotically to the transient solution of the parabolic heat-conduction equation.

The proposed modeling approach is intended to provide an alternative methodology to the expansion from the kinetic theory framework for the development of mesoscopic systems. In this case, we have developed and analyzed these systems for heat conduction; however, the proposed methodology can be used to derive mesoscopic systems for other transport phenomena, for example, those involving anomalous mass diffusion through biological systems. On the other hand, the detailed analytical treatment of the proposed systems and the analysis of their solution, that is, the expansion of the polynomial roots and analysis of the modes, is meant to provide a systematic methodology to understand their behavior and features. This analysis can be helpful to understand even more complex systems and, under proper considerations, to rationalize their design.
